# Supplementary material for: No evidence of sustained nonzoonotic Plasmodium knowlesi transmission in Malaysia from modelling malaria case data
Source: Nat Commun. 2023 Jun 1;14:2945. doi: 10.1038/s41467-023-38476-8 (PMC10235043; doi:10.1038/s41467-023-38476-8)
Supplement: Supplementary file 1 — Supplementary Information [file 41467_2023_38476_MOESM1_ESM.pdf]

Supplementary Information.

No evidence of sustained nonzoonotic *Plasmodium knowlesi* transmission in Malaysia from modelling malaria case data

Fornace, et. al.

1. Malaysian malaria surveillance data.

1.1 Geolocation and cleaning malaria surveillance data.

Malaria surveillance data included all malaria case notifications in Malaysia reported between January 2012 to December 2020. All case records included geographic data including the names of kampungs (villages) where cases were resident and locations of infections. Exact Global Positioning System (GPS) coordinates were not available for over 20% of study records, with increasing availability in later years as GPS technology was routinely implemented.

To assess the accuracy of recorded GPS coordinates, coordinates with less than 2 decimal points were first mapped onto Google Earth and locations were manually confirmed or GPS coordinates were excluded as unreliable. For each year, GPS coordinates were also imported into Quantum GIS and overlaid with administrative shapefiles for Malaysia. State and district names were extracted from administrative polygons and compared to reported states and districts listed within surveillance data. For all data where administrative units did not match, GPS coordinates were marked as unreliable and manually confirmed. For remaining GPS coordinates, data was mapped onto publicly available satellite data (Google Earth, Open Street Map) to confirm locations. All kampungs were assigned a unique code and records were manually checked to confirm any alternate spellings of names. GPS coordinates reported from the same kampung were compared to ensure locations were consistent. For records with no GPS coordinates, the centroid of the kampung was used to geolocate records. Kampung centroids were either calculated as a mean of all available records from that kampung or manually identified from satellite data, census data or by personnel familiar with the region.

To clean nonspatial malaria data, yearly surveillance data was imported into R (R Statistical Software, v 3.6.2). As the database structure changed slightly during the reporting period, data was coded with standard headings and merged. Variables not routinely collected throughout

the study period (e.g. gametocyte presence) were coded as NA for missing records. The dates of symptom onset, diagnosis, hospitalisation and notification were extracted for all records. Onset dates were excluded as unreliable if they occurred after diagnosis and/or notification dates. Due to recall bias, symptom onset dates were additionally excluded as unreliable if they occurred more than 30 days prior to diagnosis.

1.2 Surveillance data characteristics.

Between 16 December 2011 and 3 January 2021, 32,635 malaria cases were reported to the national surveillance system (Supplementary Tables 1 and 2).

Supplementary Table 1. Malaria cases by parasite species in East and West Malaysia*, number (%)

	P. falciparum	*P. knowlesi*	*P. malariae*	*P. ovale*	*P. vivax*	Mixed infections	Total
East Malaysia (Borneo)	2,196 (6.7%)	19,931 (61.1%)	1,096 (3.4%)	99 (0.3%)	2,566 (7.9%)	205 (0.6%)	26,093
West Malaysia	853 (13.0%)	3,212 (49.1%)	48 (0.7%)	20 (0.3%)	2,375 (36.3%)	34 (0.5%)	6,542

* Includes all malaria cases, including imported cases and reoccurences

Supplementary Table 2. Demographic breakdown of malaria cases, number (%)

	P. falciparum	*P. knowlesi*	*P. vivax*	All Malaria
N	3,049	23,143	4,941	32,635
Male (n, %)	2,484 (81.5%)	18,701 (80.8%)	4,209 (85.2%)	26,651 (81.7%)
Age (median, IQR)	32 (21 – 43)	36 (26 – 49)	30 (21 – 42)	35 (24 – 47)
Indigenous (n, %)	1,678 (55.0%)	23,016 (99.4%)	2,342 (47.4%)	28,149 (86.3%)

2. Serial interval estimation.

Assessing the probability of human-mosquito-human transmission of *P. knowlesi* requires estimating the timing between reported *P. knowlesi* cases from the same transmission chain. This requires estimating the duration of a series of sequential processes which need to occur

in a human-mosquito-human transmission cycle. The generation time (Tg) refers to the duration of time between an infection and the individual infecting another person [1]. The serial interval (SI), the time between clinical presentation of primary and secondary cases, is more commonly used as infection is typically unobserved [2]. While these intervals can be inferred from contract tracing data for directly transmitted diseases, these intervals can only be estimated indirectly for vector-borne diseases [3]. Additionally, there is a lack of empirical evidence on human-mosquito-human *P. knowlesi* transmission as this has only been experimentally observed once. The rapid replication cycle of *P. knowlesi* and weak evidence of adaptation to humans suggests this may differ from other nonzoonotic malaria species [4].

To estimate the generation time and serial intervals for human-mosquito-human *P. knowlesi* transmission, we used a quantitative model-based approach developed by Huber et. al [5]. This models the SI and Tg as random variables based on the sum of random variables representing the sequential steps in the transmission cycle including: the prepatent period, the human to mosquito transmission period, the extrinsic incubation period, the mosquito to human transmission period and infection to detection periods. These were parameterised using a combination of data from secondary literature and the Malaysian malaria surveillance dataset.

Within this analysis, we made two important assumptions. First, we assumed that asymptomatic human infections did not contribute to transmission. This was based on the very low parasite densities of human *P. knowlesi* infections detected during community surveys within Malaysia, likely insufficient to infect mosquitoes [6-8]. Second, we assumed that individuals became non-infectious on the date of diagnosis and treatment. This is based on the Malaysian national malaria policy of hospitalising malaria cases from diagnosis until confirmation as microscopy negative [9]. Within Malaysia, all malaria treatment is free through government healthcare providers.

1. Prepatent period (PREP). Estimates of the prepatent period for *P. knowlesi* were based on experimental *P. knowlesi* infections in people following bites from infected *An. balabacensis*, the main vector in Malaysian Borneo [10]. This was modelled as Normal(10.6 days, 1.15 days) based on data from Table 1. The time between an individual developing a patent infection and becoming symptomatic was modelled as Normal(3.5 days, 0.2 days) based on this experimental data.

2. Human to mosquito transmission period (HTMP). No data was available on the duration or timing of infectiousness of *P. knowlesi* in humans. However, multiple studies have reported *P.*

knowlesi gametocytes in people at both microscopic and submicroscopic densities [11-13]. While *P. knowlesi* gametocytes are highly synchronous in macaques [4, 14], available evidence suggests this is not the case in human infections [13]. Infectiousness to mosquitoes is dependent on the density of malaria gametocytes, with infectious individuals predominantly having gametocyte densities high enough to be detected microscopically [15]. The presence of microscopically detected *P. knowlesi* gametocytes is captured by the Malaysian surveillance data for cases reported after 2014. These data showed a clear increasing trend in the proportion of cases with observed gametocytes relative to the time since symptom onset. Using this data, we modelled Y_i, the number of infectious individuals (defined as having gametocyte densities high enough to be detected by microscopy) out of the total number of cases (n_i) as the realisation of a binomial random variable Y_i ~ Binomial(n_i, π_i). The probability an individual was infectious (π_i) was specified as logit(π_i) = $\beta_0 + \beta_1 x_i$, where β_0 is the intercept and $\beta_1 x_i$ describes the effect of days since symptom onset. This model was then used to simulate the probability and duration of infectiousness using 10,000 simulations. We assumed that no individuals had sufficient gametocyte densities to become infectious prior to developing patent malaria and, if an individual became infectious, they remained infectious until treatment. The duration of patent malaria was estimated as the sum of the time between an individual developing patent malaria and becoming symptomatic and the time between symptom onset and treatment. The time between symptom onset and treatment was fit to Malaysian surveillance data and modelled as a Gamma distribution. As there was insufficient data to estimate the probability of infectiousness two weeks past symptom onset, we assumed a constant probability of infectiousness after 15 days.

These probabilities of infectiousness were multiplied by a constant mosquito-to-human ratio estimated from empirical data on human landing catches in Sabah, Malaysia [16, 17]. While other models have used time-varying mosquito-to-human ratios ([5, 18]), Malaysia is equatorial and strong seasonal trends in mosquito densities are not typically observed. This could be expanded in future work to model geographic heterogeneities or uncertainties around this number.

3. Extrinsic incubation period (EIP). The next step in the transmission cycle occurs within the mosquito. The extrinsic incubation period (EIP) is the time between parasite transmission from an infectious human to a mosquito and the production of sporozoites within the mosquito. Previous studies have estimated the EIP for *P. knowlesi* as 10 days [17, 19]. An experimental study of *P. knowlesi* in *An. stephensi* identified the EIP as 6.8 days, however this is not a natural *P. knowlesi* vector in Malaysia [20]. To represent the uncertainty around these estimates, EIP was modelled as ~ Normal(10 days, 1 day).

4. Mosquito to human transmission period (MHTP). Following the approach described by Huber et. al, we modelled the time between the end of the EIP and the time of a subsequent human infection as a geometric random variable with probability 1 – *p*, where *p* is the constant probability of daily survival [5]. This assumes no variability in mosquito daily survival and no association between mosquito survival and probability of human infection. The probability of daily survival was estimated as 0.85 based on *An. balabacensis* data from Sabah, Malaysia [17].

5. Infection to detection period (IDP). As the SI is based on reported clinical cases, estimation of the SI also requires assessing the time between human infection by a mosquito and detection by the health facility. This was modelled as the sum of the prepatent period, time between patency and symptom onset and time between symptom onset and diagnosis, with variables parameterised based on secondary literature or malaria surveillance data as described previously.

Probabilistic descriptions of the Tg and SI were obtained by summing random variables using the approach developed by Huber et. al [5]. First, the Tg was calculated as:

$$\mathrm{Tg}(i + j + k + l) = \sum_{i}\sum_{j}\sum_{k}\sum_{l}(\mathrm{Pr}(PREP = i) \text{ x } (\mathrm{Pr}(\mathrm{HMT}P = j) \text{ x } (\mathrm{Pr}(\mathrm{EI}P = k) \text{ x } (\mathrm{Pr}(\mathrm{MHT}P = l)$$

Where *i, j, k* and *l* are dummy variables used to calculate the probability of the Tg for all combinations of *i, j, k* and *l*. The same approach was used to calculate the SI, accounting for the infection to detection periods of the primary and secondary cases:

$$\mathrm{SI}(-i + j + k) = \sum_{i}\sum_{j}\sum_{k}(\mathrm{Pr}(\mathrm{ID}P = i) \text{ x } (\mathrm{Pr}(\mathrm{GI} = j) \text{ x } (\mathrm{Pr}(\mathrm{ID}P = k)$$

Supplementary Table 3. Parameters used for SI and Tg estimation

Description	Estimate	Source
Prepatent period	~Normal(10.6 days, 1.14 days)	[21]
Time from patent infection to symptom onset	~Normal(3.5 days, 0.2 days)	[21]
Time from symptom onset until treatment	~Gamma(2.484, 0.473)	Fit to Malaysian surveillance data
Duration of infectiousness	Binomial model fit to presence/ absence of microscopically observed gametocytes from time from infection	Fit to Malaysian surveillance data, constant rate of infectiousness after day 15
Mosquito to human ratio	4	Mean biting rate reported by [22]
Probability of mosquito daily survival	0.85	[17]
Infection to detection period	Time from infection to symptom onset + Time from symptom onset to treatment	

3. Estimation of R_C

3.1 Transmission likelihood

Based on estimates of the duration of infectiousness, we fit shifted Rayleigh distributions to describe a prior distribution of possible serial intervals for nonzoonotic *P. knowlesi* transmission (Figure S1a) and *P. falciparum*/ *P. vivax* transmission (Figure S1b). Using a fixed value for the spatial parameter (δ) of 0.1, we estimated the likelihood of two cases being connected based on the geographic location and time of reporting (Figure S1). The fixed value for the spatial parameter corresponded to most cases being infected within a 10km radius of their reported residence location; this parameter was obtained based on reported travel history from case investigations reporting most individuals remaining within the same village or district prior to their diagnosis. Individuals with a history of long-range travel were classified as imported cases according to Malaysian Ministry of Health surveillance guidelines.

Supplementary Figure 1. Likelihood of two cases being part of the same transmission chain based on notification time (X axis) and geographic distance (Y axis) with priors used for a.) nonzoonotic *P. knowlesi* transmission and b.) *P. falciparum* and *P. vivax* transmission

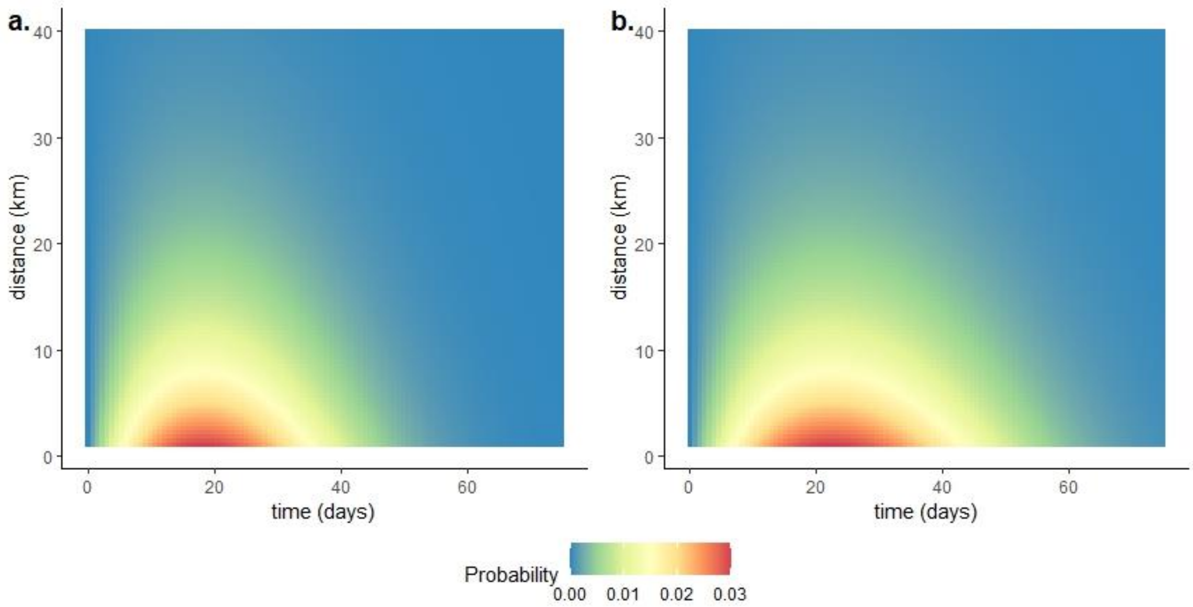

We used an adapted version of the NetRate model to estimate R_C [18, 23-25]. Data was input as a series of n infections ($I_1,\ldots I_n$) reported at times $\boldsymbol{t} = \{t_1,\ldots t_n\}$ with a binary classification of importation status. The serial interval parameters were represented by the function f_1 and the relationship between geographic location of cases and likelihood of transmission was represented by the function f_2, giving the function:

$$f\left(x_i, t_i \middle| x_j, t_j; \alpha_{i,j}, \beta\right) = f1(t_i|t_j; \alpha_{i,j}) \text{ x } f2(x_i|x_j; \beta)$$

where t is the time, x is the spatial locations, α is the transmission rate and β are the spatial parameters. The hazard is defined as the pairwise likelihood divided by the survival function as:

$$H = \frac{f\left(x_i, t_i \middle| x_j, t_j; \alpha_{i,j}, \beta\right)}{S\left(x_i, t_i \middle| x_j, t_j; \alpha_{i,j}, \beta\right)}$$

The pairwise likelihood of a case reported at time t_j and location x_j infecting a case reported at time t_i and location x_i is:

$$f\left(x_i, t_i \middle| x_j, t_j; \alpha_{i,j}, \beta\right) = \alpha(t_i - t_j - \gamma)e^{-\frac{1}{2}\alpha(t_i - t_j - \gamma)}\frac{1}{\beta}$$

With the hazard term simplifying to:

$$H(x_i, t_i | x_j, t_j; \alpha_{i,j}, \beta) = \beta\alpha(t_i - t_j - \gamma)e^{-\beta(x_i - x_j)}$$

And the survival term as:

$$S(x_i, t_i | x_j, t_j; \alpha_{i,j}, \beta) = e^{-\frac{1}{2}\alpha(t_i - t_j - \gamma)}\frac{1}{\beta}$$

Integrating the survival term over distances is equivalent to:

$$S(x_i, t_i | x_j, t_j; \alpha_{i,j}, \beta) = e^{-\frac{1}{2}\alpha(t_i - t_j - \gamma)}\frac{\sqrt{\pi}}{2\sqrt{\beta}}$$

And the hazard function is:

$$H(x_i, t_i | x_j, t_j; \alpha_{i,j}, \beta) = \frac{\alpha(t_i - t_j - \gamma)e^{-\frac{1}{2}\alpha(t_i - t_j - \gamma)}e^{-\beta(x_i - x_j)}}{e^{-\frac{1}{2}\alpha(t_i - t_j - \gamma)}\frac{\sqrt{\pi}}{2\sqrt{\beta}}}$$

Which simplifies to:

$$H(x_i, t_i | x_j, t_j; \alpha_{i,j}, \beta) = \frac{2\sqrt{\beta}\alpha(t_i - t_j - \gamma)e^{-\beta(x_i - x_j)^2}}{\sqrt{\pi}}$$

To account for potential unobserved sources of infection, we used Epsilon edges. Within this framework, a high ε value assumes the case is very likely to be from an unobserved source unless two cases have a high likelihood of being linked while a low ε assumes unobserved sources of infection are highly unlikely.

With ε, this gives:

$$f(\boldsymbol{t}, \boldsymbol{x}; \in, \boldsymbol{\beta}) = \prod_{t_i \in \boldsymbol{t}} S_0(\in_i) \prod_{I_k : t_k < t_i} S(x_i, t_i | x_j, t_j; \alpha_{i,j}, \beta)\left(H_0(\in_i) + \sum_{I_k : t_k < t_i} H(x_i, t_i | x_j, t_j; \alpha_{i,j}, \beta)\right)$$

With the objective function as:

$$minimize_{\boldsymbol{\alpha},\in} - \log f(\boldsymbol{t}, \boldsymbol{x};\ \in, \boldsymbol{\beta})\ subject\ to\ \boldsymbol{\alpha}, \in, \boldsymbol{\beta} > 0\ \ \forall i, j$$

Geolocated time series data was used to fit models with varying priors on ε, reflecting the uncertainty around the proportion of zoonotic transmission. Model fit was evaluating using the second order AIC (AICc), (Table S3). The best fitting models had no estimates of R_C greater than one for both East and West Malaysia (Figure S2).

Supplementary Table 4. Model selection statistics for *P. knowlesi* models with varying normally distributed priors on ε using a fixed value of δ=0.1 and priors of Normal(0.002, 0.001) for α

	Epsilon Priors			
Dataset	Mean	SD	Mean R_C	AICc*
East Malaysia	0.0001	0.001	0.945	-1012488877
	0.001	0.01	0.796	-1012540285
	0.01	0.1	0.431	-1012612525
	0.1	1	0.362	-1012631421
	1	1	0.074	-1012730557
West Malaysia	0.0001	0.001	0.788	-44737596
	0.001	0.01	0.450	-44748816
	0.01	0.1	0.131	-44762856
	0.1	1	0.115	-44760900
	1	1	0.015	-44768972

* Lower AICc values represent improved model fit

Supplementary Figure 2. Estimated R_C values from the best fitting models for *P. knowlesi*.

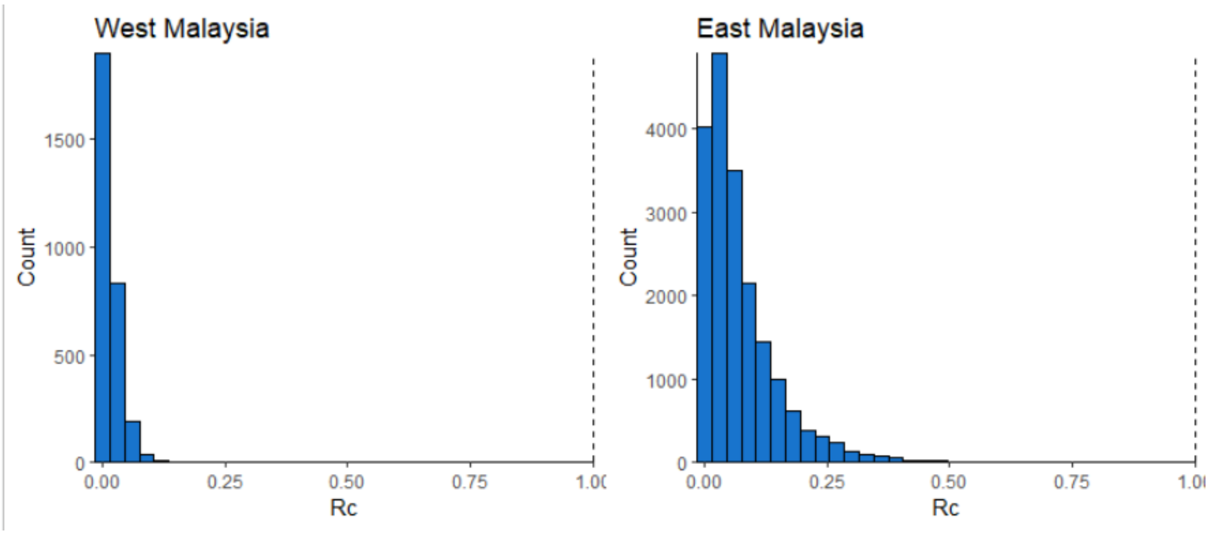

We additionally conducted sensitivity analyses for *P. falciparum* and *P. vivax* models to assess the impact of varying priors on ε while using established distributions for the temporal component. As transmission of these models is known to be nonzoonotic and extensive local transmission was documented during the study period, we excluded unlikely scenarios where all cases were imported (Supplementary Table 4).

Supplementary Table 5. Model selection statistics for *P. knowlesi* models with varying normally distributed priors on ε using a fixed value of δ=0.1 and priors of Normal(0.003, 0.001) for α for a.) *P. falciparum* and b.) *P. vivax*

a.)

	Epsilon Priors				
Dataset	Mean	SD	Mean R_C	Percent R_C > 1	AICc*
East Malaysia	0.0001	0.001	0.568	20.89%	250425034
	0.00001	0.01	0.568	20.95%	250425274
	0.000001	0.1	0.565	20.90%	250487594
West Malaysia	0.0001	0.001	0.386	9.14%	6401555
	0.00001	0.0001	0.101	10.13%	6401460
	0.000001	0.00001	0.099	9.91%	6403864

b.)

	Epsilon Priors				
Dataset	Mean	SD	Mean R_C	Percent R_C > 1	AICc*
East Malaysia	0.0001	0.001	0.524	20.31%	652347120
	0.00001	0.01	0.547	21.19%	652347568
	0.000001	0.1	0.558	21.47%	652347952
West Malaysia	0.0001	0.001	0.230	8.61%	219812878
	0.00001	0.0001	0.246	8.92%	219813166
	0.000001	0.00001	0.250	9.13%	219842030

3.2 Spatiotemporal models of $\mathrm{R_C}$

To visualise the spatial and temporal distributions of *P. falciparum* and *P. vivax* cases with R_C estimates > 1, we fit geostatistical models using Integrated Nested Laplace Approximation (INLA) in R statistical software. For each species, we classified R_C estimates into binary classes based on whether R_C estimates were greater than 1. We resampled all data to 5 km^2 grid cells and calculated the total number of *P. falciparum* or *P. vivax* cases per grid cell ($m_{i,t}$); *i* = 1…n; *t* = 1…n; where *i* indexes location and *t* indexes year. For each species, we fit separate models for the probability of a malaria case leading to onward transmission (R_C > 1) was modelled as:

$$Y_{i,t} \sim \text{Binomial}\left(m_{i,t}, \pi_{i,t}\right)\right)$$

Where $\pi_{i,t}$ is the probability of a malaria case having an R_C estimate over 1 with the linear predictor for the binomial model specified as:

$$\text{logit}\left(\pi_{i,t}\right) = \beta_0 + w_i + e_t$$

Where β_0 is the intercept, w_i is the spatial effect and e_t is the temporal effect. Candidate models including the temporal effect as a random effect, temporally structured random walk models or autoregressive models were evaluated using the Deviance Information Criteria (DIC). The final model included the temporal effect as a temporally structured random walk model of order 2 [26]. The spatial effect w_i was modelled as a Matern covariance function implemented using the stochastic partial differential equations approach. All models used 1,000 samples to estimate posterior probabilities and were visualised in R. As there were no R_C estimates above 1 for the best fitting *P. knowlesi* models, models were only fit for *P. falciparum* and *P. vivax*. Mean and maximum R_C estimates per village per year are included below in Figure S3.

Supplementary Figure 3. R_C estimates for *P. knowlesi* per village per year, including a) mean R_C estimates and b) maximum R_C estimates (areas with highest probability of nonzoonotic *P.knowlesi* transmission)

a.)

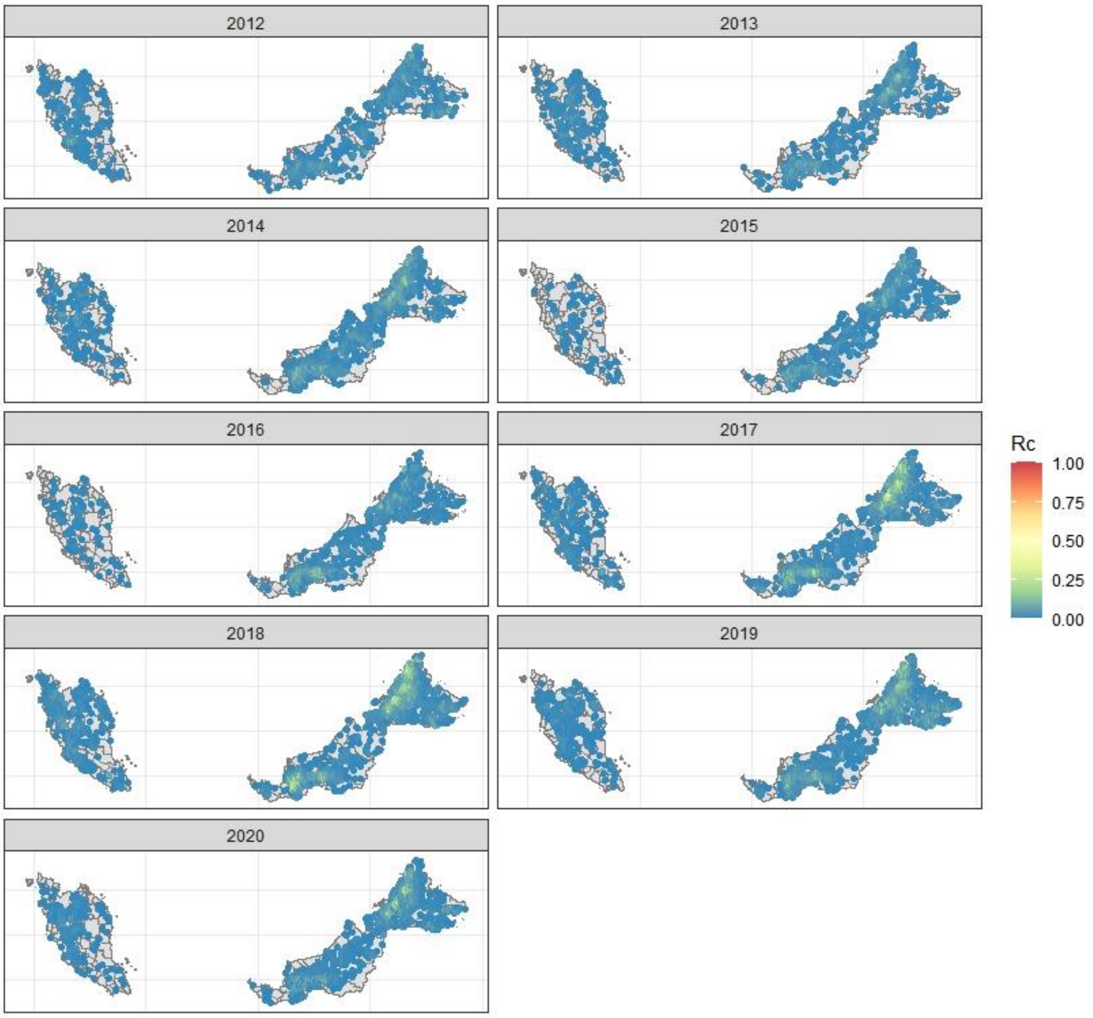

b.)

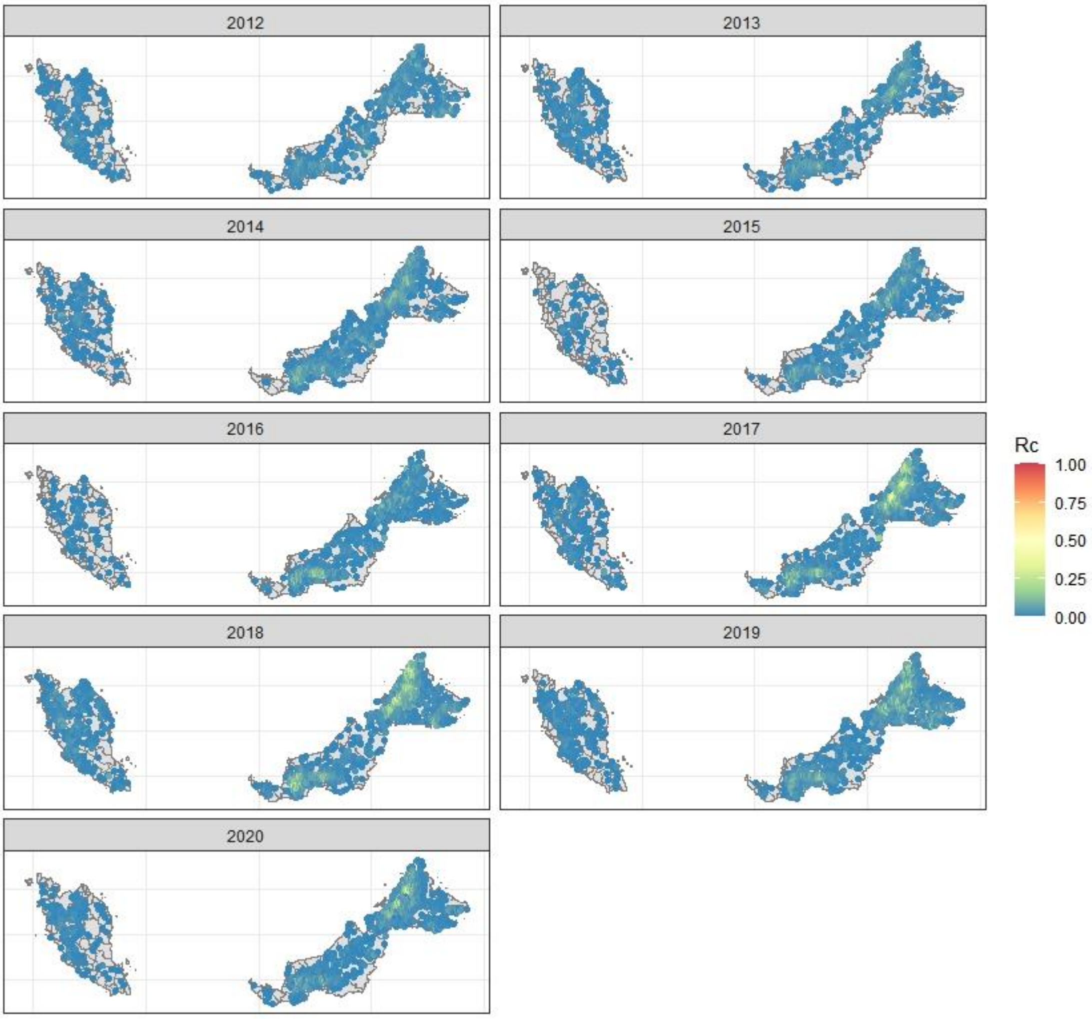
2012
2013
2014
2015
2016
2017
2018
2019
2020
Rc
1.00
0.75
0.50
0.25
0.00

1. Champredon, D. and J. Dushoff, *Intrinsic and realized generation intervals in infectious-disease transmission.* Proc Biol Sci, 2015. **282**(1821): p. 20152026.
2. Fine, P.E., *The interval between successive cases of an infectious disease.* Am J Epidemiol, 2003. **158**(11): p. 1039-47.
3. Park, S.W., D. Champredon, and J. Dushoff, *Inferring generation-interval distributions from contact-tracing data.* J R Soc Interface, 2020. **17**(167): p. 20190719.
4. Hawking, F., M.J. Worms, and K. Gammage, *24 and 48 hour cycles of malaria parasites in the blood; their purpose, production and control.* Transactions of the Royal Society of Tropical Medicine and Hygiene, 1968. **82**(6): p. 731-765.
5. Huber, J.H., et al., *Quantitative, model-based estimates of variability in the generation and serial intervals of Plasmodium falciparum malaria.* Malar J, 2016. **15**(1): p. 490.
6. Fornace, K.M., et al., *Environmental risk factors and exposure to the zoonotic malaria Plasmodium knowlesi across Northern Sabah, Malaysia: a cross-sectional survey.* Lancet Planet Health, 2019. **3**(4): p. E179-E186.
7. Fornace, K.M., et al., *Exposure and infection to Plasmodium knowlesi in case study communities in Northern Sabah, Malaysia and Palawan, The Philippines.* PLoS Neglected Tropical Diseases [electronic resource], 2018. **12**(6): p. e0006432.
8. Fornace, K.M., et al., *Asymptomatic and Submicroscopic Carriage of Plasmodium knowlesi Malaria in Household and Community Members of Clinical Cases in Sabah, Malaysia.* J Infect Dis, 2016. **213**(5): p. 784-7.
9. Ministry of Health Malaysia, *Management guidelines of malaria in Malaysia*. 2014, Vector Borne Disease Sector, Disease Control Division, Ministry of Health, Malaysia: Putra Jaya.
10. Chin, W., et al., *Experimental mosquito-transmission of Plasmodium knowlesi to man and monkey.* American Journal of Tropical Medicine & Hygiene, 1968. **17**(3): p. 355-8.
11. Grigg, M.J., et al., *Artesunate-mefloquine versus chloroquine for treatment of uncomplicated Plasmodium knowlesi malaria in Malaysia (ACT KNOW): an open-label, randomised controlled trial.* Lancet Infect Dis, 2016. **16**(2): p. 180-8.
12. Maeno, Y., et al., *Plasmodium knowlesi and human malaria parasites in Khan Phu, Vietnam: Gametocyte production in humans and frequent co-infection of mosquitoes.* Parasitology, 2017. **144**(4): p. 527-535.
13. Lee, K.S., J. Cox-Singh, and B. Singh, *Morphological features and differential counts of Plasmodium knowlesi parasites in naturally acquired human infections.* Malar J, 2009. **8**: p. 73.
14. Anderios, F., A. Noorrain, and I. Vythilingam, *In vivo study of human Plasmodium knowlesi in Macaca fascicularis.* Exp Parasitol, 2010. **124**(2): p. 181-9.
15. Bradley, J., et al., *Predicting the likelihood and intensity of mosquito infection from sex specific Plasmodium falciparum gametocyte density.* Elife, 2018. **7**.
16. Fornace, K.M., et al., *Local human movement patterns and land use impact exposure to zoonotic malaria in Malaysian Borneo.* Elife, 2019. **8**.
17. Wong, M.L., et al., *Seasonal and Spatial Dynamics of the Primary Vector of Plasmodium knowlesi within a Major Transmission Focus in Sabah, Malaysia.* PLoS Negl Trop Dis, 2015. **9**(10): p. e0004135.
18. Routledge, I., et al., *Estimating spatiotemporally varying malaria reproduction numbers in a near elimination setting.* Nat Commun, 2018. **9**(1): p. 2476.
19. Imai, N., et al., *Transmission and control of Plasmodium knowlesi: a mathematical modelling study.* PLoS Negl Trop Dis, 2014. **8**(7): p. e2978.
20. Hawking, F., et al., *Transmission of Plasmodium knowlesi by Anopheles stephensi.* Trans R Soc Trop Med Hyg, 1957. **51**(5): p. 397-402.
21. Chin, W., et al., *Experimental mosquito-transmission of Plasmodium knowlesi to man and monkey.* Am J Trop Med Hyg, 1968. **17**(3): p. 355-8.

22. Fornace, K.M., et al., *Local human movement patterns and land use impact exposure to zoonotic malaria in Malaysian Borneo.* eLife, 2019. **8**(10): p. 22.
23. Routledge, I., et al., *Tracking progress towards malaria elimination in China: Individual-level estimates of transmission and its spatiotemporal variation using a diffusion network approach.* PLoS Comput Biol, 2020. **16**(3): p. e1007707.
24. Routledge, I., H.J.T. Unwin, and S. Bhatt, *Inference of malaria reproduction numbers in three elimination settings by combining temporal data and distance metrics.* Sci Rep, 2021. **11**(1): p. 14495.
25. Gomez-Rodriguez, M., D. Balduzzi, and B. Scholkpf, *Uncovering the temporal dynamics of diffusion networks.* Proceedings of the 28th International Conference on Machine Learning, 2011.
26. Lindgren, F. and H. Rue, *Bayesian Spatial Modelling with R-INLA.* Journal of Statistical Software, 2015. **63**(19).
