# Supplementary material for: No evidence of sustained nonzoonotic Plasmodium knowlesi transmission in Malaysia from modelling malaria case data
Source: Nat Commun. 2023 Jun 1;14:2945. doi: 10.1038/s41467-023-38476-8 (PMC10235043; doi:10.1038/s41467-023-38476-8)
Supplement: Supplementary file 3 — Reporting Summary [file 41467_2023_38476_MOESM3_ESM.pdf]

nature portfolio

Corresponding author(s): Kimberly Fornace

Last updated by author(s): Apr 20, 2023

Reporting Summary

Nature Portfolio wishes to improve the reproducibility of the work that we publish. This form provides structure for consistency and transparency in reporting. For further information on Nature Portfolio policies, see our Editorial Policies and the Editorial Policy Checklist.

Statistics

For all statistical analyses, confirm that the following items are present in the figure legend, table legend, main text, or Methods section.

n/a	Confirmed	
☐	☒	The exact sample size (*n*) for each experimental group/condition, given as a discrete number and unit of measurement
☒	☐	A statement on whether measurements were taken from distinct samples or whether the same sample was measured repeatedly
☐	☒	The statistical test(s) used AND whether they are one- or two-sided<br>*Only common tests should be described solely by name; describe more complex techniques in the Methods section.*
☐	☒	A description of all covariates tested
☐	☒	A description of any assumptions or corrections, such as tests of normality and adjustment for multiple comparisons
☐	☒	A full description of the statistical parameters including central tendency (e.g. means) or other basic estimates (e.g. regression coefficient) AND variation (e.g. standard deviation) or associated estimates of uncertainty (e.g. confidence intervals)
☒	☐	For null hypothesis testing, the test statistic (e.g. *F*, *t*, *r*) with confidence intervals, effect sizes, degrees of freedom and *P* value noted<br>*Give P values as exact values whenever suitable.*
☐	☒	For Bayesian analysis, information on the choice of priors and Markov chain Monte Carlo settings
☐	☒	For hierarchical and complex designs, identification of the appropriate level for tests and full reporting of outcomes
☒	☐	Estimates of effect sizes (e.g. Cohen's *d*, Pearson's *r*), indicating how they were calculated

Our web collection on statistics for biologists contains articles on many of the points above.

Software and code

Policy information about availability of computer code

Data collection	Data was collected from the Malaysian National Malaria Surveillance Programme and cleaned and geolocated using R (v 4.1) and Quantum GIS (v 3.24).
Data analysis	Data was analysed in R statistical software (v4.1) and Quantum GIS (v3.24). Code and simulated datasets are available at: https://github.com/IzzyRou/spatial_rcs with a full description of simulation studies available at: https://doi.org/10.1038/s41598-021-93238-0

For manuscripts utilizing custom algorithms or software that are central to the research but not yet described in published literature, software must be made available to editors and reviewers. We strongly encourage code deposition in a community repository (e.g. GitHub). See the Nature Portfolio guidelines for submitting code & software for further information.

Data

Policy information about availability of data

All manuscripts must include a data availability statement. This statement should provide the following information, where applicable:

- Accession codes, unique identifiers, or web links for publicly available datasets
- A description of any restrictions on data availability
- For clinical datasets or third party data, please ensure that the statement adheres to our policy

As surveillance datasets contain identifiable health information, data is only available following approval of the relevant ethics committees in Malaysia and the UK and the Malaysian Ministry of Health. Requests to access datasets should be directed to Kimberly.Fornace@lshtm.ac.uk

Human research participants

Policy information about studies involving human research participants and Sex and Gender in Research.

Reporting on sex and gender	Self-reported gender was used in descriptive analyses only.
Population characteristics	Data on age, gender, residence location, admission dates and clinical information on malaria diagnoses were used in these analyses. Full breakdowns of these characteristics are included in the supplementary information and this includes all malaria cases reported between 2012- 2020 in Malaysia.
Recruitment	No recruitment was conducted as this was an analysis of secondary surveillance data from reported clinical cases.
Ethics oversight	National Medical Research Ethics Committee of Malaysia, Malaysian Ministry of Health; London School of Hygiene and Tropical Medicine Research Ethics Committee

Note that full information on the approval of the study protocol must also be provided in the manuscript.

Field-specific reporting

Please select the one below that is the best fit for your research. If you are not sure, read the appropriate sections before making your selection.

☒ Life sciences ☐ Behavioural & social sciences ☐ Ecological, evolutionary & environmental sciences

For a reference copy of the document with all sections, see nature.com/documents/nr-reporting-summary-flat.pdf

Life sciences study design

All studies must disclose on these points even when the disclosure is negative.

Sample size	Sample sizes were not calculated as this included all reported malaria cases within the study time period (2012-2020) to examine possible chains of transmission within this entire dataset.
Data exclusions	Data was excluded if it could not be geolocated (e.g. contained no address). Out of 32,635 cases, 2,117 records were excluded for this reason
Replication	Code and simulation studies were conducted to support reproducibility of results.
Randomization	No randomisation was included as this was an analysis of all available secondary data representing the total dataset of malaria cases reported in Malaysia from 2012- 2020.
Blinding	No blinding was used as all cases were included in the analyses to evaluate the likelihood of nonzoonotic transmission and estimate case reproduction numbers. Inference methods were used to assess the likelihood of nonzoonotic transmission.

Reporting for specific materials, systems and methods

We require information from authors about some types of materials, experimental systems and methods used in many studies. Here, indicate whether each material, system or method listed is relevant to your study. If you are not sure if a list item applies to your research, read the appropriate section before selecting a response.

Materials & experimental systems

n/a	Involved in the study	
☒	☐	Antibodies
☒	☐	Eukaryotic cell lines
☒	☐	Palaeontology and archaeology
☒	☐	Animals and other organisms
☒	☐	Clinical data
☒	☐	Dual use research of concern

Methods

n/a	Involved in the study	
☒	☐	ChIP-seq
☒	☐	Flow cytometry
☒	☐	MRI-based neuroimaging
